# A181 HIGHER CUMULATIVE HISTOLOGIC INFLAMMATORY BURDEN SCORE IS ASSOCIATED WITH THE RISK OF DEVELOPMENT OF COLORECTAL NEOPLASIA IN ULCERATIVE COLITIS

**DOI:** 10.1093/jcag/gwac036.181

**Published:** 2023-03-07

**Authors:** D Graff, C Hernandez-Rocha, K Borowski, J Stempak, J Conner, M S Silverberg

**Affiliations:** 1 Zane Cohen Centre for Digestive Diseases, Lunenfeld-Tanenbaum Research Institute, Sinai Health System; 2 Division of Gastroenterology, Sinai Health System, University of Toronto; 3 Department of Pathology & Laboratory Medicine, Sinai Health Systems, Toronto, Canada

## Abstract

**Background:**

Ulcerative colitis (UC) patients have an elevated risk of colorectal neoplasia (CRN). Younger age at diagnosis, extent of colitis, and longer duration of colitis, as well as increased severity, which is a component of the cumulative inflammatory burden score (CIB), have been associated with the development of CRN. CIB was developed based on a large cohort of UC patients from St. Mark’s Hospital (UK) but needs further validation in independent cohorts.

**Purpose:**

We analyzed the association between higher histologic CIB and development of CRN in longstanding UC patients.

**Method:**

A matched case-control cohort of UC patients with at least 8 years of disease duration was analyzed at Mount Sinai Hospital. Patients with primary sclerosing cholangitis were excluded. Cases consisted of UC patients with colitis-associated neoplasia defined as indefinite for dysplasia (IND), low-grade dysplasia (LGD), high-grade dysplasia (HGD), or colorectal cancer (CRC). Each case was matched to two controls by age at disease onset, disease duration, and histological extent of colitis. Histologic reports obtained by colonoscopy were reviewed and histological activity was assessed as quiescent/normal (0), mild (1), moderate (2), and severe (3). The colonic area with the higher score was utilized and the CIB was calculated by summing each score and multiplying it by the interval of surveillance. A mean CIB (mCIB) was also calculated for each patient dividing the CIB by the number of colonoscopies. Continuous variables including CIB scores and mCIB scores were summarized as median and interquartile range (IQR) and differences between groups were compared by Mann-Whitney test.

**Result(s):**

Fifty-four UC patients were analyzed with 18 having CRN (6 CRC, 2 HGD, 3 LGD and 7 IND) and 36 controls without CRN. The clinical characteristics of the total cohort, cases and controls are depicted in the Table. Median age at last colonoscopy assessed was 45 years (36-55) and 40.7% were female. The median age at onset of UC was 23 years (19-37) and median duration of UC was 16 years (11-23). All patients had extensive histologic colonic disease. There were no differences between cases and controls in interval of surveillance evaluated (7.5 vs 7.8 years, p = 0.7) and median number of colonoscopies with histologic assessment (4 vs 4, p =0.6). Cases with CRN had significantly higher CIB (11.4 vs 7.9, p = 0.02) and mCIB (2.9 vs 2.0, p = 0.02) compared to controls.

**Image:**

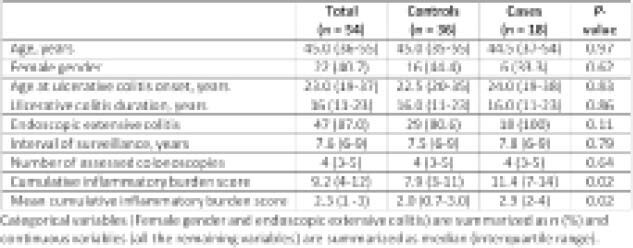

**Conclusion(s):**

The histologic CIB score is associated with an increased risk of developing CRN in UC patients with similar age at onset of disease, disease duration and colitis extent. Given CIB reflects the severity of histologic inflammation over the years, treatment strategies to improve histologic inflammation could reduce the incidence of CRN in UC.

**Please acknowledge all funding agencies by checking the applicable boxes below:**

None

**Disclosure of Interest:**

None Declared

